# Retrospective Analysis of 234 Nasopharyngeal Carcinoma Patients with Distant Metastasis at Initial Diagnosis: Therapeutic Approaches and Prognostic Factors

**DOI:** 10.1371/journal.pone.0108070

**Published:** 2014-09-23

**Authors:** Lei Zeng, Yun-Ming Tian, Ying Huang, Xue-Ming Sun, Feng-Hua Wang, Xiao-Wu Deng, Fei Han, Tai-Xiang Lu

**Affiliations:** 1 Department of Radiation Oncology, Sun Yat-Sen University Cancer Center, State Key Laboratory of Oncology in South China, Collaborative Innovation Center of Cancer Medicine, Guangzhou, PR China; 2 Department of Medical Oncology, Sun Yat-Sen University Cancer Center, State Key Laboratory of Oncology in South China, Collaborative Innovation Center of Cancer Medicine, Guangzhou, PR China; 3 Department of Radiation Oncology, Jiangxi Cancer Hospital, Nanchang, PR China; Northwestern University Feinberg School of Medicine, United States of America

## Abstract

**Purpose:**

The purpose of this retrospective study was to identify the independent prognostic factors and optimize the treatment for nasopharyngeal carcinoma (NPC) patients with distant metastasis at initial diagnosis.

**Methods:**

A total of 234 patients referred between January 2001 and December 2010 were retrospectively analyzed. Among the 234 patients, 94 patients received chemotherapy alone (CT), and 140 patients received chemoradiotherapy (CRT). Clinical features, laboratory parameters and treatment modality were examined with univariate and multivariate analyses.

**Results:**

The median overall survival (OS) time was 22 months (range, 2-125 months), and the 1-year, 2-year, 3-year overall survival rates were 82.2%, 51.3% and 34.1%. The overall response and disease control rates of metastatic lesions after chemotherapy were 56.0% and 89.8%. The factors associated with poor response were karnofsky performance score (KPS) <80, liver metastasis, lactate dehydrogenase (LDH)>245 IU/L, and number of chemotherapy cycles <4. The 3-year OS of patients receiving CRT was higher than those receiving CT alone (48.2% vs. 12.4%, p<0.001). Subgroup analysis showed that significantly improved survival was also achieved by radiotherapy of the primary tumor in patients who achieved complete remission (CR)/partial remission (PR) or stable disease (SD) of metastatic lesions after chemotherapy. Significant independent prognostic factors of OS were KPS, liver metastasis, levels of LDH, and multiple metastases. Treatment modality, response to chemotherapy and chemotherapy cycles were also associated with OS.

**Conclusion:**

A combination of radiotherapy and chemotherapy seems to have survival benefits for selected patients with distant metastases at initial diagnosis. Clinical and laboratory characteristics can help to guide treatment selection. Prospective randomized studies are needed to confirm the result.

## Introduction

Nasopharyngeal carcinoma (NPC) is a common epithelial malignancy in southern China. The highest incidence has been reported in Guangdong province, where the rate is approximately 20 per 100,000 people per year [1], [2]. Radiotherapy alone has become the standard treatment for early stage disease, and chemoradiotherapy for the advanced NPC [3]. Biologically different from other squamous cell cancers of the head and neck, approximately 95% of these cases were undifferentiated carcinomas with the highest incidence of distant metastases [4], [5]. Once metastasis is diagnosed, the overall survival of patients is very poor after palliative chemotherapy. Furthermore, patients with distant metastasis at initial diagnosis had been demonstrated with a significantly shorter survival when compared with those with subsequent metastases [6]–[10].

However, patients with distant metastasis at initial diagnosis do not behave in a uniform manner. It is hence not surprising to see significantly variable results between studies of similar therapeutic approaches in patients with metastatic NPC [11], [12]. Although palliative chemotherapy has been demonstrated as the most effective way with high objective response rates, recurrence frequently occurs after chemotherapy ceases. However, the application of radiotherapy of the primary tumor remains controversial because of their short life expectancy and radiation-induced complications [11]–[13].

Therefore, determining the prognostic factors of survival outcomes in NPC patients with distant metastasis at initial diagnosis could help to select those patients who would most benefit from comprehensive treatment including radiotherapy of the primary tumor by retrospectively analyzing patients’ clinical characteristics, treatment modalities and survival. These results might contribute to management of treatment and exploration of avenues of further research.

## Materials and Methods

### Patients and selection criteria

Between January 2001 and December 2010, 271 NPC patients presenting with distant metastases at initial diagnosis were referred to our cancer center. The selection criteria were as follows: (1) pathologically confirmed NPC in the nasopharynx, (2) diagnosis of distant metastasis based on physical examination and imaging, (3) receiving at least one anti-cancer treatment including the chemotherapy and the radiotherapy, (4) complete follow-up and clinical data, including laboratory and imaging data. Patients with other malignancies or unstable cardiac disease requiring treatment were excluded. Of the 271 NPC patients, 37 patients were excluded from the survival analysis, including 14 cases because of missing clinical data and 23 cases because of refusing any treatment, leaving 234 patients for evaluation. The clinicopathological data of the 234 patients are presented in Tables 1, 2, 3.

**Table 1:** Clinical characteristics.

Characteristics	N(%)
Gender	
Female	32(14)
Male	202(86)
Age (years)	
<48	116(50)
≥48	118(50)
Karnofsky performance score (KPS)	
<80	30(13)
≥80	204(87)
Histology	
WHO Type 2	8(3)
WHO Type 3	226(97)
Bony metastasis	
Present	157(67)
Absent	77(33)
Liver metastasis	
Present	75(32)
Absent	159(68)
Lung metastasis	
Present	36(15)
Absent	198(85)
Distant nodal metastasis	
Present	27(12)
Absent	207(88)
No. of metastatic sites	
Single	52(22)
Multiple	182(78)

**Table 2:** Laboratory characteristics.

Characteristics	N(%)
Haemoglobin (g/L)	
<120	29(12)
≥120	205(88)
Lactate dehydrogenase (LDH) (IU/L)	
≤245	154(66)
>245	80(34)
Alkaline phosphatase (ALP) (IU/L)	
≤110	182(78)
>110	52(22)
VCA-IgA	
Negative	13(5)
Positive	221(95)

**Table 3:** Treatment characteristics.

Characteristics	N(%)
Treatment modality	
Chemotherapy alone	94(40)
Chemoradiotherapy	140(60)
Chemotherapy regimen	
Cisplatin+fluorouracil	124(53)
Paclitaxel+cisplatin	110(47)
Chemotherapy response	
Progression of disease	24(10)
Stable disease*	79(34)
Complete remission+Partial remission†	131(56)
Chemotherapy cycles	
1–3 cycles	74(32)
≥4 cycles	160(68)

*52 patients received RT to primary lesions and 27 patients did not received RT;

† 88 patients received RT to primary lesions and 43 patients did not received RT.

Ethical Review Committee of Sun Yat-Sen University Cancer Center has approved the project. Written consent was given by the patients to be stored in the hospital database.

### Pre-treatment evaluation

All patients had a pre-treatment evaluation including complete history, physical examination, hematology and biochemistry profiles, Epstein-Barr virus (EBV) serology, chest radiographs, sonography of abdomen, whole-body bone scan and magnetic resonance imaging (MRI) of head and neck regions. A titre of more than 1∶20 was considered to be positive for the VCA-IgA antibodies as adopted in previous study on the marker [9]. Patients were evaluated according to the 2002 American Joint Committee on Cancer (AJCC) TNM stages.

### Treatment

The treatment modalities were determined according to the experience of our center and the acceptance of the patients. Radiotherapy of the primary tumor was generally administrated to those patients who achieved disease control of the metastatic lesions after chemotherapy. It was also administered to reduce serious symptoms caused by the primary tumor that affected the quality of life. Among the 234 patients, 94 patients received chemotherapy alone (CT), and 140 patients received chemoradiotherapy (CRT).

All the patients were treated with cisplatinum-based chemotherapy. The median number of cycles of chemotherapy was 5 (range, 1–14).

Among of the patients who received RT, 116 (82.9%) were treated with conventional techniques, 20 (14.3%) underwent intensity-modulated radiotherapy (IMRT) and 4 underwent three-dimensional conformal radiotherapy (3D-CRT). Details regarding the RT techniques have been previously reported [14]–[15]. One hundred and seventeen patients received a radiation dose ≥66 Gy and 23 patients underwent a dose <66 Gy. The median dose was 70 Gy (range, 40–78 Gy).

Fifty-five patients received local therapy to metastases, including 39 patients received radiotherapy to bone lesion (30–66Gy/10-33f), 10 received radiofrequency ablation (RFA) and 3 received interventional embolization of liver lesions, and 3 received surgery of lung lesions.

### Treatment evaluations and follow up

Imaging of the metastasis was performed after every two courses of chemotherapy, and then every 3 months during follow-up. Objective response was measured according to the Response Evaluation Criteria in Solid Tumors (RECIST). The evaluation of bone metastasis was based on the imaging findings of re-calcification shown in CT and the decreased concentration in the whole bone scanning and the clinical evidence of the pain relief.

Patients were followed up by direct telecommunication mean or by checking the clinic attendance records. The overall survival (OS) was defined as the duration from the date of diagnosis to the date of death from any cause or the censoring of the patient at the date of the last follow-up. The median follow-up for the whole was 22 months (range, 2-125).

### Statistical analysis

Statistical analysis was performed using SPSS 13.0 package. Overall survival (OS) was analyzed using the Kaplan-Meier method and was compared using the log-rank test. Univariate and multivariate analysis were performed using the Cox proportion hazards model. The multivariate analyses were undertaken with both forward and backward stepwise procedures for identifying variables correlated with overall survival. Covariates included patients’ characteristics (Karnofsky performance score, gender and age), laboratory parameters (hemoglobin, lactate dehydrogenase, alkaline phosphatase and the EBV serology), metastatic features (extension and response to chemotherapy) and treatment approaches (number of chemotherapy cycles, radiotherapy of the primary tumor and local therapy of metastases). Furthermore, the relationship of response to chemotherapy and various factors was tested by logistic regression model. A two-tailed P-value <0.05 was considered statistically significant.

## Results

### Treatment response and overall survival

One hundred and fifty-four patients had been dead by the final evaluation date. The main cause of death was progression died of metastatic lesions, which occurred in 137/154 (89.0%) patients; 15/154 (9.7%) patients died of local failure and 2/154 (1.3%) die of cardiac disease. The median OS time was 22 months (range, 2-125 months), and the 1-year, 2-year, 3-year overall survival rates were 82.2%, 51.3% and 34.1%, respectively.

Of the 234 patients, 10/234 (4.3%) achieved complete response (CR) of metastatic lesions, 121/234 (51.7%) achieved partial response (PR), 79/234 (33.8%) had stable disease (SD) and 24/234 (10.2%) had progressive disease (PD). The overall response and disease control rates were 56.0% and 89.8%, respectively. Logistic regression analysis showed that the following factors were significantly associated with poor response to chemotherapy (PD+SD): KPS <80 (P = 0.016); liver metastasis (P = 0.001); LDH>245 IU/L (P = 0.023); and number of chemotherapy cycles <4 (P<0.001).

### Toxicities

Two of the patients died of treatment-related toxicity including one with severe infection caused by the grade IV leucopenia and one with the hepatic failure during chemotherapy exhibited. In total, 45.3% developed grade III–IV leucopenia or neutropenia and 16.7% exhibited grade II–III toxicity with vomiting and nausea. Among the patients receiving RT, the most significant toxicity was the grade 3/4 mucositis with a rate of 40.5%, and the skin reaction with a rate of 25.0%. All patients completed the full course of RT.

### Univariate analysis and Multivariate analysis

The result of univariate analysis and multivariate analysis are summarized in Table 4 and Table 5. The negative prognostic factors in the univariate analysis for OS were as follows include KPS<80 (P<0.001), LDH>245 (P<0.001), ALP>110 (P<0.001), Liver metastasis (HR = 2.204, P<0.001), and Multiple metastases (P<0.001). CT alone (P<0.001), Chemotherapy cycles<4 (P = 0.001), Poor response to chemotherapy (P<0.001), and Without local therapy to metastatic lesions (P<0.001) were also associated with poor OS in the univariate analysis.

**Table 4:** Univariate analysis of variables correlated with overall survival.

Characteristic	Univariate Analysis	
	P	HR (95% CI)
Gender, men vs women	0.096	1.536(0.927–2.545)
Age, <48 vs ≥48	0.787	0.957(0.698–1.314)
KPS, <80 vs ≥80	<0.001^a^	4.712(3.018–7.358)
Liver metastasis, yes vs no	<0.001^a^	2.204(1.598–3.039)
Lung metastasis, yes vs no	0.377	0.819(0.525–1.276)
Bone metastasis, yes vs no	0.754	0.948(0.681–1.321)
Distant nodal metastasis, yes vs no	0.800	1.069(0.636–1.798)
Number of involved site,>1 vs 1	<0.001^a^	2.648(1.678–4.178)
Haemoglobin, <120 vs ≥120	0.933	1.021(0.624–1.672)
Serum LDH,>245 vs ≤245	<0.001^a^	2.554(1.843–3.538)
Serum ALP,>110 vs ≤110	<0.001^a^	2.124(1.497–3.014)
VCA-IgA, Positive vs Negative	0.370	0.734(0.374–1.443)
Local therapy to metastases, no vs yes	<0.001^a^	2.565(1.657–3.970)
Treatment modality, CT vs CRT	<0.001^a^	3.058(2.202–4.247)
Response to chemotherapy, PR+CR		Baseline
SD	<0.001^a^	2.251(1.583–3.202)
PD	<0.001^a^	6.455(3.876–10.735)
Chemotherapy cycles, <4 vs ≥4	0.001^a^	1.783(1.280–2.484)

HR: hazard ration; CI: confidence interval; CT: Chemotherapy CRT: Chemoradiotherapy; PD: Progression of disease; SD: Stable disease; PR: Partial remission; CR: Complete remission;

a Statistically significant.

**Table 5:** Multivariate analysis of variables correlated with overall survival.

Variables	HR(95%CI)	P
Clinical and Laboratory Characteristic		
KPS, <80 vs ≥80	4.077(2.481–6.700)	<0.001^a^
Liver metastasis, yes vs. no	1.652(1.140–2.393)	0.008^a^
Number of involved site, >1 vs 1	2.106(1.288–3.444)	0.003^a^
Serum LDH, >245 vs ≤245	1.686(1.187–2.395)	0.004^a^
Treatment Characteristic		
Treatment modality, CT vs CRT	2.066(1.440–2.964)	<0.001^a^
Chemotherapy cycles, <4 vs ≥4	1.748(1.223–2.499)	<0.001^a^
Response to chemotherapy, PR+CR	Baseline	
SD	2.338(1.591–3.437)	<0.001^a^
PD	3.370(1.947–5.833)	<0.001^a^

HR: hazard ration; CI: confidence interval; CT: Chemotherapy CRT: Chemoradiotherapy; PD: Progression of disease; SD: Stable disease; PR: Partial remission; CR: Complete remission;

a Statistically significant.

**Figure 1:**
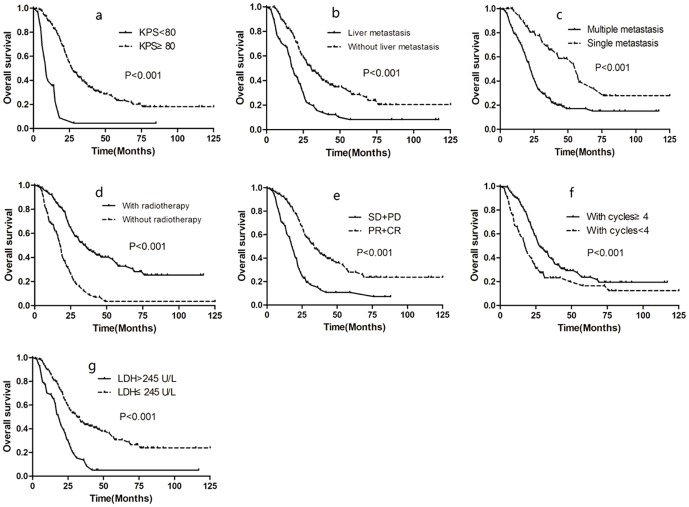
Overall survival rates according to KPS (a), liver metastasis (b), number of metastatic site (c), radiotherapy of primary tumor (d), response to chemotherapy (e), number of cycles of chemotherapy (f) and LDH (g).

The multivariate analysis show that the significant prognostic factors for poor survival were KPS<80 (HR = 4.077, P<0.001), LDH>245 (HR = 1.748, P = 0.004), Liver metastasis (HR = 1.652, P = 0.008), and Multiple metastases (HR = 2.106, P = 0.003). CT alone (HR = 2.066, P<0.001), Chemotherapy cycles<4 (HR = 1.748, P<0.001), and Poor response to chemotherapy(PD group, HR = 6.455, P<0.001; SD group, HR = 2.251, P<0.001) were also associated with poor OS in the multivariate analysis. Patients with good performance status (KPS≥80) survived longer than those with poor performance status (3-year OS: 37.9% vs. 4.4%). Patients with normal LDH level had a better survival than those with high LDH level (3-year OS: 44.7% vs. 13.7%). The 3-year OS rate for patients with liver metastasis was poorer than those without liver metastasis (14% vs. 45.7%). Patients with single metastasis had a better survival than those with multiple metastases (the 3-year survival rates: 65.8% vs. 25.9%). Furthermore, the therapy related factors were also associated with OS. The 3-year OS rate for patients receiving chemotherapy cycles <4 was poorer than those receiving chemotherapy cycles ≥4 (23.2% vs. 39.1%). The 3-year survival of patients receiving CRT was 48.2%, better than those receiving CT alone with only 12.4%. Patient with response to chemotherapy of metastatic lesions also show better survival with 3-year OS rate of 38.0% for patients with PR or CR, and 14.2% for patients with SD, and none for patients with PD. These results are shown in Figure 1.

**Figure 2:**
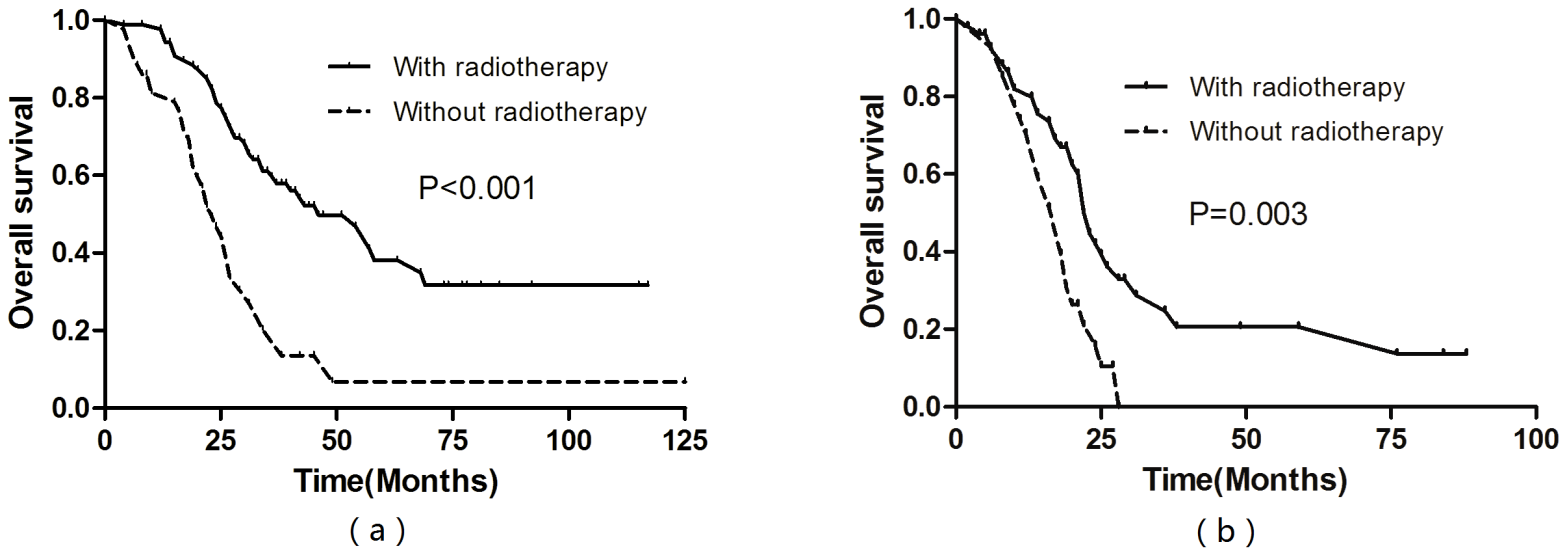
Overall survival rates for patients who achieved CR or PR after chemotherapy of metastatic lesions (a), for patients who achieved SD after chemotherapy of metastatic lesions (b).

For patients who achieved CR or PR after chemotherapy of metastatic lesions, multivariate analysis showed that radiotherapy of the primary tumor was an independently significant favorable prognostic factor (HR = 0.435, P = 0.001). Significantly improved survival was achieved by radiotherapy of the primary tumor in these patients (3-year OS rate 59.6% vs. 20.3%, P<0.001, Figure 2a). For patients who achieved SD after chemotherapy of metastatic lesions, multivariate analysis also showed that radiotherapy of the primary tumor was an independently significant favorable prognostic factor (HR = 0.363, P = 0.001). Significantly improved survival was achieved by radiotherapy of the primary tumor in these patients (3-year OS 24.7% vs. 0%, P = 0.003, Figure 2b).

## Discussion

For patients presenting with distant metastases at initial diagnosis, the optimal treatment strategy remains a subject of debate [11]–[13]. The benefits of systemic chemotherapy have been demonstrated in some studies and considered as the only possibly curative option. Platinum-based combination regimen achieves high response rates and is the most widely used regimen [11], [16], [17]. For the number of cycles of chemotherapy was an independent factor associated with survival, it was important for patients receive a sufficient number of cycles. However, it was still uncertain regarding the optimal cycles of chemotherapy. In a retrospective study involving 20 long-term disease-free survivors with metastatic NPC reported by Fandi et al. [11], the results showed that approximately six cycles of chemotherapy were required. In the current study, the cut-off point of number of cycles was evaluated by the Receiver operating characteristic (ROC) and the patients with at least four cycles of chemotherapy had a significantly better survival than those with less than four cycles. The results indicated the importance of sufficient chemotherapy for patients with metastatic NPC. However, owing to the retrospective nature of this study, it was still hard to determine the optimal cycles of chemotherapy. Furthermore, the response of metastatic lesions to chemotherapy was demonstrated as a significant predictor of OS. The overall response rate (CR and PR) after chemotherapy was 56.0%, and poor response was associated with KPS <80, liver metastasis, LDH>245 IU/L and number of chemotherapy cycles <4, suggesting that these factors could be potential predictors of treatment response. The response of metastatic lesions to chemotherapy also plays a key part in the consideration of the treatment choice. The results indicated that patients with CR or PR were recommended for a more progressive treatment as this could significantly improve survival.

In the clinical practice, the most controversial issue for NPC patients initially with metastases was the application of radiotherapy to the primary tumor for the uncertain indications in the guideline of NCCN (National Comprehensive Cancer Network), which posed great challenge for the oncologists [12], [13]. It was often considered as inappropriate to give a prolonged course of radiotherapy to patients with stage IVC NPC because of their short life expectancy and serious late complications in the past era. However, due to the improvements in radiation techniques and increasing efficacy of platinum-based combination regimen, some studies show that the local control of primary tumor following the radiotherapy would improve the quality of life and contribute to prolonged survival. In a retrospective analysis of 125 NPC patients initially with metastases reported by Yeh et al. [13], the 2-year OS rate was 24.0% when they received radiotherapy alone when compared to 10% in those who received chemotherapy alone, and it also showed that the local control of the primary tumor improved the quality of life because of the reduced necrosis, bleeding and severe headaches. In the current study, the application of radiotherapy after chemotherapy was a positive factor associated with survival. The 3-year OS of patients receiving radiotherapy after chemotherapy was up to 48.2%, significantly higher than those receiving chemotherapy alone with only 12.4%. However, the survival benefit may be also related to the selection for radiotherapy. Therefore, it was very important to select the patients who would most benefit from the radiotherapy. In the subgroup analysis, we found the radiotherapy could significantly improve the survival of patients who achieved the CR or PR of metastatic lesions after chemotherapy with a 3-year OS rate of 59.6%. Even though for patients who achieved SD after chemotherapy of metastatic lesions, significantly improved survival was achieved by radiotherapy of the primary tumor in (3-year OS 24.7% vs. 0%, P = 0.003).

These findings indicated that excellent local control may help reduce the tumor burden and the risks of death caused by progression of primary tumor, especially for the patients with CR/PR or SD of metastatic lesions after chemotherapy. Furthermore, the improvements of radiation technique such as the application of Intensity-modulated radiotherapy (IMRT) may further improve the treatment benefit.

Part of our results were consistent with those reported by Toe et al. [6], liver metastasis was associated with poor survival. In the current study, the 3-year OS rate of patients with liver metastasis was only about 14.0%, significantly poorer than those with other metastasis included the lung, bone or distant nodal metastasis with a 3-year OS rate of 43.7%. In the retrospective analysis of 379 NPC patients with subsequent metastases reported by Hui et al. [7], the lung metastasis alone was demonstrated as a positive factor of survival and long-term survival was possible for those patients. The reason for poor survival of liver metastasis may relate to the rich blood supply of liver and the low rate of the response to chemotherapy. Furthermore, the patients with single metastasis exhibited the excellent survival with 3-year OS rate of 65.8%, while only 25.9% for those patients with multiple metastases. It may be the sub-group of long-term survival after aggressive approach to treatment.

Elevated levels of LDH also demonstrated as a negative prognostic factor, which may be associated with large tumor burden, tumor extension and high risk of metastasis [18]–[20]. Serum LDH levels twice normal levels are rarely seen in loco-regional disease but are commonly observed in NPC patients with liver metastasis or multiple organ metastases. Studies have found that NPC patients with elevated baseline LDH levels were more likely to develop liver metastasis after treatment. In the study of Jin et al. [20], elevated LDH levels were reported in over 55.0% of patients with metastatic NPC, the relative risk to die increased with LDH>245 IU/L by the factor 1.8. In our study, the 3-year OS rate of patients with normal level of LDH was about 47.7%, significantly higher those with elevated LDH levels with a 3-year OS rate of 13.7%. More than 60% of patients with liver metastasis had elevated levels of LDH. Furthermore, elevated LDH was also associated with poor response of metastatic lesions to chemotherapy. Pretreatment serum level of LDH may be a potential predictor.

This retrospective analysis has several weaknesses. First, the circulating EBV DNA load has been demonstrated as an independent prognostic factor in disseminated NPC [21]. However, only small part of patients’ EBV DNA data was collected in our study, therefore we had excluded the factor to avoid the potential bias. Second, treatment modality has an impact on survival outcome in patients with disseminated NPC at initial diagnosis. Since the treatment modalities were selected according to the physician’s policy of practice in our study, it is inevitable to cause selection bias when we identify prognostic factors for patients with distant metastases at initial diagnosis.

## Conclusion

In this study we identified some negative prognostic factors for patients with distant metastases at initial diagnosis which included poor performance status, elevated levels of LDH, liver metastasis and multiple metastases. We also found that chemotherapy alone, chemotherapy cycles<4 and poor response to chemotherapy were associated with poor OS. It can help to select the appropriate patient for more progressive treatment of a combination of chemotherapy and radiotherapy. Long-term survival is possible for patients with less negative prognostic factors. Prospective randomized studies are needed to optimize treatment strategy.
